# Regional Differences and Similarities in the Brain Transcriptome for Mice Selected for Ethanol Preference From HS-CC Founders

**DOI:** 10.3389/fgene.2018.00300

**Published:** 2018-08-28

**Authors:** Alexandre M. Colville, Ovidiu D. Iancu, Denesa R. Lockwood, Priscila Darakjian, Shannon K. McWeeney, Robert Searles, Christina Zheng, Robert Hitzemann

**Affiliations:** ^1^Department of Behavioral Neuroscience, Oregon Health & Science University, Portland, OR, United States; ^2^Department of Medical Informatics and Clinical Epidemiology, Oregon Health & Science University, Portland, OR, United States; ^3^Integrated Genomics Laboratory, Oregon Health & Science University, Portland, OR, United States; ^4^Knight Cancer Institute, Oregon Health & Science University, Portland, OR, United States

**Keywords:** RNA-Seq, collaborative cross, nucleus accumbens shell, central nucleus of amygdala (CeA), prelimbic cortex, network analysis

## Abstract

The high genetic complexity found in heterogeneous stock (HS-CC) mice, together with selective breeding, can be used to detect new pathways and mechanisms associated with ethanol preference and excessive ethanol consumption. We predicted that these pathways would provide new targets for therapeutic manipulation. Previously (Colville et al., 2017), we observed that preference selection strongly affected the accumbens shell (SH) genes associated with synaptic function and in particular genes associated with synaptic tethering. Here we expand our analyses to include substantially larger sample sizes and samples from two additional components of the “addiction circuit,” the central nucleus of the amygdala (CeA) and the prelimbic cortex (PL). At the level of differential expression (DE), the majority of affected genes are region-specific; only in the CeA did the DE genes show a significant enrichment in GO annotation categories, e.g., neuron part. In all three brain regions the differentially variable genes were significantly enriched in a single network module characterized by genes associated with cell-to-cell signaling. The data point to glutamate plasticity as being a key feature of selection for ethanol preference. In this context the expression of *Dlg2* which encodes for PSD-93 appears to have a key role. It was also observed that the expression of the clustered protocadherins was strongly associated with preference selection.

## Introduction

Beginning with Lewohl et al. (2000) there are now more than 200 studies using some form of genome-wide profiling to examine the relationships among alcohol effects, excessive alcohol consumption and the brain transcriptome. Contet (2012) reviewed the existing literature and noted that the genes associated with the risk of excessive consumption and/or the effects of excessive consumption had regionally specific effects on gene expression. Subsequent studies have confirmed and extended the “region" effect (e.g., Melendez et al., 2012; Osterndorff-Kahanek et al., 2015; Smith et al., 2016; Mulligan et al., 2017). It is important to note that these studies also by and large confirmed earlier observations (e.g., Kimpel et al., 2007) that regional differences in gene expression are generally far greater than the effects of treatment, strain or line (e.g., Mulligan et al., 2017). From a somewhat different perspective we have also observed that the regional transcriptional network signature is largely independent of genetic diversity (Iancu et al., 2010).

In the current study we explore at the regional level how selection for ethanol preference affects the transcriptome. The regions compared (nucleus accumbens shell [SH], central nucleus of the amygdala [CeA], and prelimbic cortex [PL]) are components of the addiction circuit (Koob and Volkow, 2010, 2016). A previous study (Dhaher et al., 2008) suggested that the CeA but not the SH has a more significant role in preference (2-bottle choice) consumption. The short-term selection of the High and Low ethanol preference lines from heterogeneous stock-collaborative cross (HS-CC) founders has been described elsewhere (Colville et al., 2017). After three generations of bidirectional selection, the difference in the ethanol preference ratio was 0.49 vs. 0.15 in the High and Low lines, respectively. Sixty-five percent of the High females and 37% of the High males had a preference ratio of >0.5 compared with 6.5% of the Low females and 2.3% of the Low males. The HS-CC founders (formed from five laboratory and three wild-derived strains) provide substantially more genetic diversity than would be available in F_2_ intercrosses or HS animals formed solely from inbred laboratory mouse strains (Roberts et al., 2007). It is estimated that the HS-CC founder strains encompass >90% of *Mus musculus* genetic diversity (Churchill et al., 2004).

Colville et al. (2017) used RNA-Seq to examine how High/Low line selection affected the SH transcriptome. The data analysis emphasized the effects of selection on gene networks. Networks were constructed using the weighted gene coexpression network analysis (WGCNA) (Zhang and Horvath, 2005). Selection targeted one of the network coexpression modules that were significantly enriched in genes associated with receptor signaling activity, including *Chrna7, Grin2a, Htr2a*, and *Oprd1*. Connectivity in the module as measured by changes in the hub nodes was significantly reduced in the low preference line. The current study expands on these observations by asking what features are regionally specific or non-specific. For this purpose, sample sizes have been substantially increased from Colville et al. (2017) to insure the high quality of network structures across brain regions (see Langfelder et al., 2011).

## Materials and Methods

### Husbandry

The short term selection lines (Colville et al., 2017) were obtained from the colony at the Portland VA Medical Center, an AAALAC approved facility. All procedures were in accordance with the VA Institutional Animal Care and Use Committee and were performed according to NIH Guidelines for the Care and Use of Laboratory Animals. Mice were maintained at 21 ± 1°C in plastic cages (19 cm × 31 cm × 13 cm) on Eco-Fresh bedding (Absorption Corp.) with tap water and Purina 5001 chow (PMI Nutrition International, Brentwood, MO, United States) given *ad libitum*. Pups were weaned and housed with same-sex litter mates at postnatal day 21.

### Selection

Selection details are found in Colville et al. (2017). Briefly, HS-CC founders (Iancu et al., 2010) were selected for breeding based on their preference for 10% ethanol vs. water. Beginning with 200 founders, the 20 males and 20 females with the highest preference values were paired, with brother-sister matings avoided, to create a “High” preference line; similarly, the 40 mice with the lowest preference scores were paired to create a “Low” line. ∼200 pups from each generation were weaned and tested at adulthood as above for three subsequent generations; active selection concluded at S_3_. S_4_ alcohol-naive pups were used for genetic analyses.

### Dissection of Tissue and Extraction of RNA

At 8 weeks of age, naive S_4_ mice, balanced for sex and line, were euthanized, the brains removed and immediately frozen on dry ice. Frozen brains were sliced in 55 micron coronal sections on a freezing microtome at −13°C and slices containing the nucleus accumbens, the amygdala, and the medial prefrontal cortex were mounted on PEN slides. Mounted slices were lightly thionin-stained under RNAse-free conditions and dehydrated in increasing concentrations of ethanol diluted in RNAse free water (50, 70, 95, and 100%) for 30 s each and then air-dried. The shell of the accumbens (SH), the CeA and the PL were dissected bilaterally on a Leica LMD-6000 using known anatomical landmarks (Franklin and Paxinos, 2008). Dissected tissue was processed with the ARCTURUS PicoPure kit. RNA quality was assessed using the Caliper LabChip GX and RNA Quality Scores (RQS). Only samples with RQS scores of >7 and >100 ng of total RNA were used for library formation. Sample numbers were as follows: SH-71; CeA-67; and PL-54. For reasons that were not clear, the percentage of extractions from the PL for high quality RNA was significantly lower.

### RNA-Seq

Library formation (polyA+, stranded) and sequencing were all performed according to Illumina’s specifications at the OHSU Massively Parallel Sequencing Shared Resource. Libraries were multiplexed six per lane, yielding approximately 25–30 million totals read per sample. FastQC was used for quality checks on the raw sequence data. Sequence data were then aligned using STAR [Spliced Transcripts Alignment to a Reference (Dobin et al., 2013)] allowing for a maximum of three mismatches per 100 bp read. For all samples >85% of the reads uniquely aligned. Using the featureCounts suite (Liao et al., 2014), reads were aligned to known genomic features to generate counts at the gene level. Gene expression data were imported into the R application environment; upper-quartile normalization was performed using the edgeR Bioconductor package (Robinson et al., 2010). The gene read density threshold for inclusion in the network analyses was an average of >1 count per million (CPM). Network connectivity for coexpression was calculated as described elsewhere (Colville et al., 2017). The expression data have been deposited to NCBI’s Gene Expression Omnibus^1^.

### Differential Expression (DE), Differential Variability (DV), and Differential Wiring (DW) Analyses

Differential expression was determined using edgeR, with the option of “tagwise” dispersion. Adjustment for multiple comparisons was performed using the SGOF procedure (de Uña-Alvarez, 2012). The threshold for significance was set at adjusted *p*-value < 0.05, although for module enrichment we utilized unadjusted *p*-values < 0.01. For gene differentially variable (DV), we utilized the “var.test” procedure in the R “stats” package; the threshold for significance was also set at adjusted *p*-value < 0.05. To mitigate the computational load for detecting differential wiring (DW), we restricted the search to Pearson correlations between individual genes that differed by >0.5. This general procedure has been used to quantify network rewiring in both genomic (Gill et al., 2010) and neural imaging studies (Hosseini et al., 2012). Using this procedure, we identified for each gene, the number of changed edges and then inquired as to whether some genes had a disproportionately high number of changing edges. For the latter, the binomial test was used with the following parameters. The average incidence of changing edges (the rate of the binomial test) was computed by dividing the number of changing edges (*p* < 0.01) by the total number of network edges. The number of trials (for each gene) was equal to the number of edges. The number of “successes” was equal to the number of changing edges.

### Coexpression Network Construction

The coexpression network was constructed by means of the WGCNA (Langfelder and Horvath, 2008; Iancu et al., 2012). We started by constructing adjacency network matrices independently for each region by computing the Pearson correlation between all gene pairs. These values were raised to a power β = 6 for all regions, which was chosen such that the network approaches a scale-free structure (exponential distribution of node connectivity).

Given that biological mechanisms of network components are best captured by the most connected genes, we restricted the size of the network to genes that were in the top 80% with regards to connectivity. This also reduces the overall network size and decreases the computational load while preserving scale-free topology. The resulting networks contained ∼6,500 genes in the three networks (see **Supplementary Tables**).

We clustered the adjacency matrices utilizing average linkage and the WGCNA cuttreeHybrid function with the following parameters: cutHeight = 0.9995, minClusterSize = 100, and deepSplit = 4. The resulting clusters (denoted as modules) are uniquely identified by arbitrarily chosen colors which are independently generated for each brain region.

To determine the extent to which modules are preserved across brain regions we employed two complementary procedures. First we utilized the WGCNA modulePreservation function to check whether modules detected in one region show increased coexpression/connectivity in the other regions, recognizing that they might be distributed across different modules even if preserved. A second measure of module preservation was computed based on the gene overlap between all module pairs in all three regions, which is denoted as tabulation-based module preservation in the modulePreservation WGCNA function (Langfelder et al., 2011).

### Coexpression Module Characterization

Module enrichment in DE, DV, and DW genes was used to assess the effects of selection on network structure. We considered a module “enriched” based on overlap between module genes and DE/DV/DW genes, using Fisher’s exact test with Bonferroni correction for number of modules. The Gorilla algorithm (Eden et al., 2009) was used to provide a visual representation of GO annotation enrichment. To implement a ranking procedure we integrated differential network results at the module and gene summarization level into a comprehensive gene screening procedure. Modules enriched in gene or edge changes were the primary focus of further annotations. At the individual gene level, we focused on module hubs with normalized intramodular connectivity above 0.8 (see Colville et al., 2017; Iancu et al., 2018).

## Results

### Summary of Gene Expression Data in the SH, CeA, and PL

The average gene expression levels across the three brain regions are presented in **Supplementary Table S1**; data are provided for the Ensembl annotated “genes” (*N* = 42,282). In all regions approximately 15,000 “genes” met the threshold of one CPM reads. Genes showing at least a 10-fold difference in expression between two regions are also found in **Supplementary Table S1**. Some expected examples include the high expression of *Adora2, Penk*, and *Drd2* in the CeA and SH and the high expression of *Bdnf* and *Cck* in the PL.

### Gene Coexpression Networks

**FIGURE 1:**
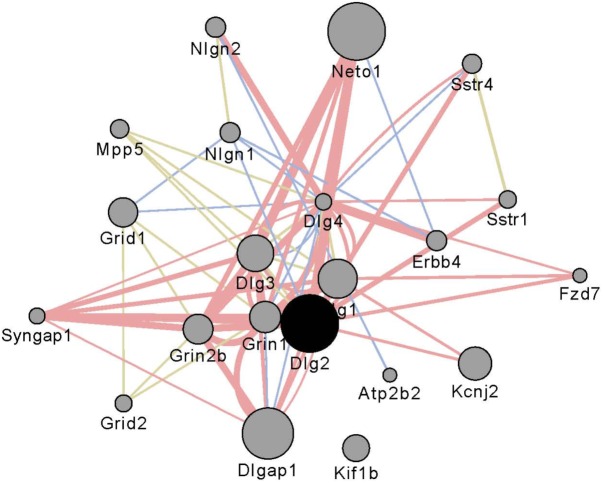
Interaction partners for *Dlg2* extracted using Gene Mania (Warde-Farley et al., 2010) which was accessed as a Cytoscape plugin with default settings. Depicted are top 20 genes related to *Dlg2* through physical interactions, colocalizations, or sharing protein domains. *Dlg2* which encodes for PSD93, interacts with a number of genes and gene products associated with glutamate receptor activity including *Dlg4, Syngap1, Neto, Grin1, Grin2b, Dlgap1*, and *Dlg3*.

Gene networks were constructed using the WGCNA as described elsewhere (Colville et al., 2017). Initially all genes meeting the expression criteria of one CPM were entered into the analysis using a consensus module approach (Iancu et al., 2010). The number of genes in each network was then culled to include only those genes that contribute >80% of the total network connectivity. It was these reduced sets of genes (∼6,500/region) that were entered into subsequent analyses. Modules were color coded arbitrarily within or across regions. **Supplementary Table S2** also provides annotation for which network modules were significantly enriched in genes associated with neurons, astrocytes, and oligodendrocytes (Cahoy et al., 2008). We investigated the interaction subnetwork of *Dlg2*, a gene affected by selection and well-connected in the network. Utilizing the GeneMANIA (Warde-Farley et al., 2010) software as implemented in the associated Cytoscape (Shannon et al., 2003) plugin, we found a number of non-transcriptional mechanisms by which *Dlg2* interacts with other members of the glutamate family (**Figure 1**).

### Module Preservation and Affected Gene Module Distribution Across Regions

Utilizing the tabulation-based module preservation procedure, we quantified the extent to which modules overlap across regions. The vast majority of modules were preserved across regions (Z summary > 2), as described in Langfelder et al. (2011). There were a few exceptions: the CeA modules cyan, greenyellow, midnightblue, and yellow were either not preserved or only mildly preserved (2 < Z summary < 3) in both SH and PL. The SH modules grey60 and lightgreen were not preserved in either CeA or PL; additionally SH lightcyan was not preserved in CeA. The PL module midnightblue was not preserved in the CeA. The rest of the modules were either preserved (2 < Z summary < 10) or in most cases highly preserved (Z summary > 10). These results illustrate that transcriptional network organization is overall preserved across brain regions, although the strength of interaction varies.

**FIGURE 2:**
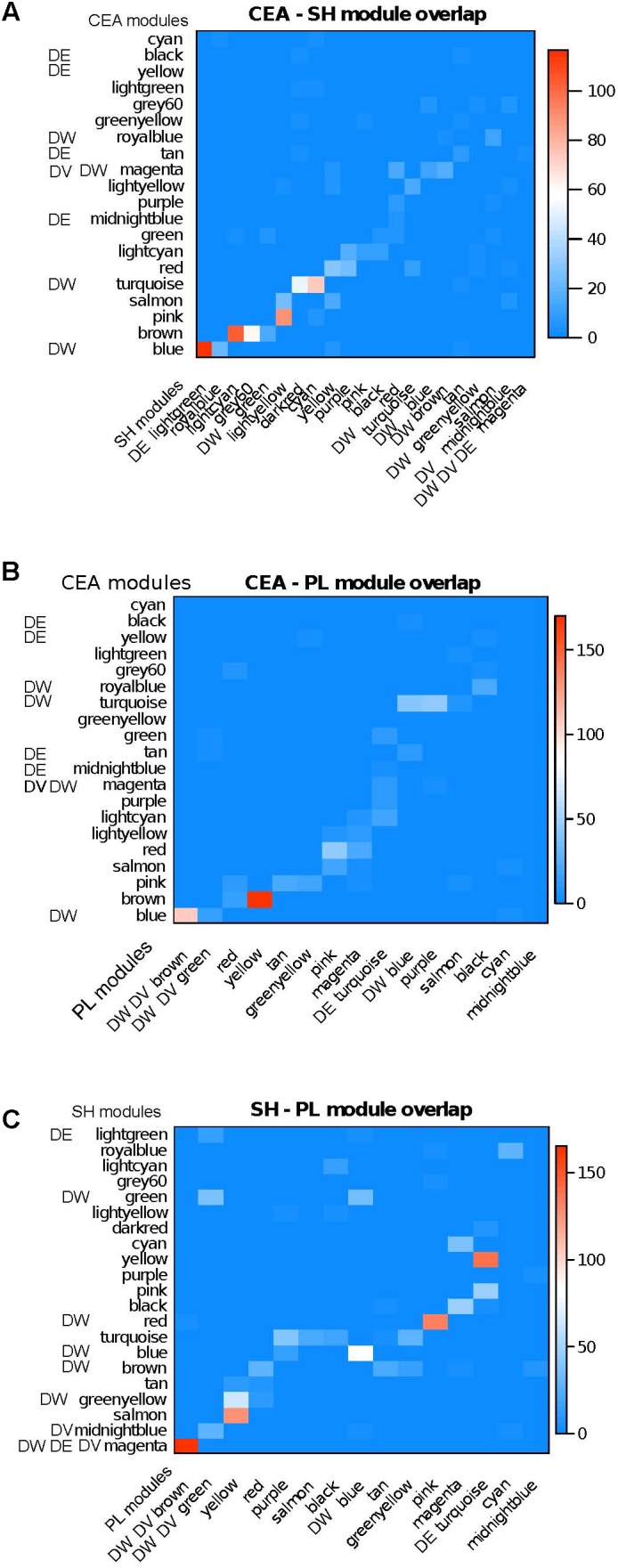
Module overlap across brain regions together with enrichment of modules in DE/DV/DW genes. Color from blue to red is proportional to –log10(p) of overlap between module membership (Fisher exact test). A majority of modules have strong counterparts across regions. The affected modules also have an affected counterpart in a majority of cases, although we also find region specific affected modules. **(A)** CeA – SH; **(B)** CeA – PL; **(C)** SH – PL.

We also utilized a complementary module preservation measure which is tabulation-based and uses the Fisher exact test. This measure evaluates whether the intersection of two modules originating from different brain regions is greater than what can be expected by chance. We found that in most cases each module has one or at most 2–3 counterparts in different brain regions (**Figure 2**). When overlaying the DE/DV/DW information on the module overlap, a complex picture emerges. We have examples of counterpart modules being affected across region, for example the DW CeA blue module having a very strong counterpart in the DE SH lightgreen module (**Figure 2A**). Another example of concordance across regions includes the DE, DV, and DW SH magenta module having a strong counterpart in the DV, DW PL brown module (**Figure 2C**). The clearest example of lack of concordance is the DE, DV, DW SH magenta module with no counterpart in the CeA (**Figure 2A**).

### Differential Expression (DE) Across Regions

**FIGURE 3:**
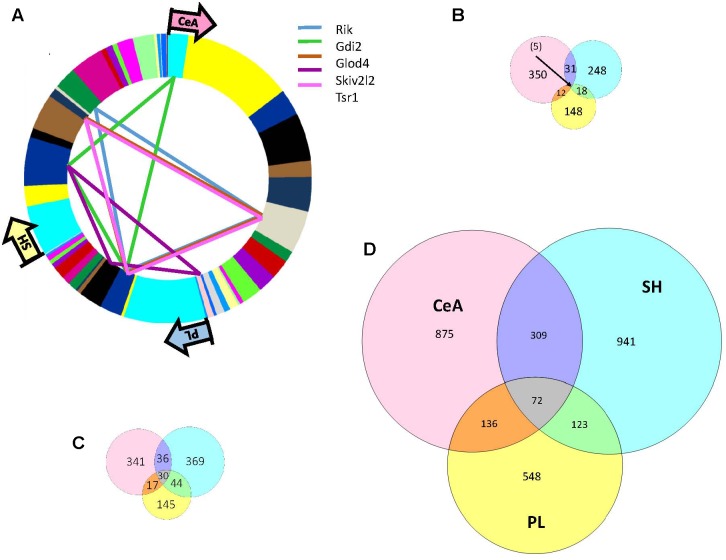
Overlap of selection associated DE, DV, and DW genes across three brain regions: CeA, SH, and PL. **(A)** As indicated in the Venn diagram **(B)** there was only 5 DE genes common to all three brain regions: *5730455P16Rik,Gdi2, Skiv2, Tsr1*, and *Glod4*. The region and module distribution of these genes is illustrated. The greatest overlap was between the CeA and SH (*N* = 31). Only annotation of the CeA DE genes revealed a significant enrichment in GO categories that included neuron part, structural constituent of myelin sheath and axon ensheathment. Genes in the neuron part category included *Adora1, Chrna4, Crhr1, Drd1a,Gabbr2, Gabrd, Gal, Htr1a, Htr2a, Htr7, Pde1b, Reln, Syt2*, and *Tac1.* **(C)** Overlap of selection associated DV genes across three brain regions: CeA, SH, and PL. There were 30 significant DV genes common to all three brain regions and this grouping was significantly enriched (FDR < 3 × 10^–3^) in genes associated with the GO annotation of cell to cell signaling. Genes with this GO annotation included *Dlg2, Egr3, Gabbr2, Lnpep, Pcdhgb2, Pcdhac2, Sstr4*, and *Syt10*. The significant DV genes unique to each brain region also showed an enrichment in genes associated with cell to cell signaling. **(D)** Overlap of selection associated DW genes across three brain regions: CeA, SH, and PL. There were 72 significant DW genes common to all three brain regions and this grouping was significantly enriched (FDR < 5 × 10^–3^) in genes associated with the GO annotation of post-synapse. Genes with this GO annotation included *Chrna7, Als2, Pppir9a, Strn, Kcna4, Kif1a*, and *Slc1a2*. Genes showing unique DW to each of the three brain regions were enriched in genes associated with the GO annotation synapse or synapse part.

There were 398, 302, and 183 genes showing significant (adjusted *p*-value < 0.05) DE between the High and Low selected lines in the CeA, SH, and PL, respectively (**Supplementary Table S3**). The overlap in DE is illustrated in **Figure 3**. Only five genes (*5730455P16Rik, Gdi2, Skiv2, Tsr1*, and *Glod4*), all with increased expression in the High line, showed common DE. The overlap for DE was highest between the SH and CeA (*N* = 31). Genes in all the overlapping categories are listed in **Supplementary Table S3**. Only one gene showed a difference in the direction of DE between regions; *Doc2b* showed increased/decreased expression in the High line (PL vs. SH). GO annotation of the CeA DE genes revealed a significant enrichment in genes associated with the neurononal component (FDR < 3 × 10^−5^), structural constituent of myelin sheath (FDR < 4 × 10^−3^) and axon ensheathment (FDR < 7 × 10^−3^) (**Supplementary Table S4**). Genes in the neuron part category included *Adora1, Chrna4, Crhr1, Drd1a, Gabbr2, Gabrd, Gal, Htr1a, Htr2a, Htr7, Pde1b, Reln, Syt2*, and *Tac1*. The CeA DE genes were significantly (corrected *p* < 8 × 10^−7^) enriched in the yellow network module (**Supplementary Table S2**). The yellow module was enriched in annotations associated with plasma membrane (FDR < 5 × 10^−4^), regulation of nervous system development (FDR < 4 × 10^−4^) and structural constituent of myelin sheath (FDR < 9 × 10^−3^; **Supplementary Table S4**). The average relative intramodular connectivity (full scale – 0.0 to 1.0) for the yellow module DE genes in the Low and High lines was 0.30 and 0.31, respectively (see **Supplementary Table S3**). Five of the 109 DE yellow module genes were hub nodes (relative connectivity >0.80 in either the High or Lines or both lines). These genes were *Rbm24, Dock10, Prkcd, Rap1gap*, and *Spg2.*

There was no significant enrichment in any GO annotation for the DE genes in the SH. Similarly, this group of genes was not enriched in any of the SH network modules. The average relative intramodular connectivity for these DE genes in the Low and High lines was 0.41 and 0.39, respectively.

There was no significant enrichment in any GO annotation for the DE genes in the PL. This group of genes was however, significantly enriched (*p* < 4 × 10^−5^) in the PL turquoise network module. This module was enriched in genes with the Rho GTPase binding annotation (FDR < 6 × 10^−3^). The average relative intramodular connectivity for these DE genes in the Low and High lines was 0.43 and 0.32, respectively (*p* < 0.003). Five of the 83 DE turquoise module genes were hub nodes. These genes were *Mapk7, Pcgf2, Leng2, Col5a3*, and *Pabpn1.*

### Differential Variability (DV) Across Regions

There were 424, 479, and 236 genes showing significant (adjusted *p*-value < 0.05) DV between the High and Low selected lines in the CeA, SH, and PL, respectively (**Supplementary Table S5**). The overlap in DV is illustrated in **Figure 3C**. Thirty genes were common to all three regions and this grouping was significantly (FDR < 3 × 10^−3^) enriched in genes associated with cell-to-cell signaling. Genes with this GO annotation included *Dlg2, Egr3, Gabbr2, Lnpep, Pcdhgb2, Pcdhac2, Sstr4*, and *Syt10*. The overlap in genes (*N* = 44) between the PL and SH showed a significant enrichment in genes with the GO annotation of neuron projection (FDR < 6 × 10^−3^). Genes with this GO annotation included *Bace1, Cpeb3, Fzd3, Igf1r, Igsf9, Kcna3, Kcnb1, Kcnma1, Slc8a1, Sv2c, Tenm1*, and *Tenm3.* In all three regions the direction of the DV was High line > > Low line.

GO annotation of the CeA DV genes revealed a significant enrichment in genes associated with cell-to-cell signaling (FDR < 3 × 10^−4^; **Supplementary Table S7**). Genes in this category and not already noted above included *Chat, Gabrg3, Glra3, Gpr88, Ntrk2, Pten, Sdcbp*, and five additional protocadherins. The CeA DV genes were significantly (1 × 10^−8^) enriched in a single network module, blue. Annotations for the blue module included synaptic membrane (FDR < 5 × 10^−2^) and cell-to-cell signaling (FDR < 2 × 10^−7^; **Supplementary Table S6**). The blue module contained most of the cell-to-cell signaling genes noted above and included *Chrm5, Chrna7, Chrnb2, Grid1, Grik3, Grin2a, Grin2b, Htr5a*, and *Sv2c;* the module was also associated with 16 protocadherin genes. For the blue module DV genes, intramodular connectivity was significantly different between the Low and High lines (0.32 vs. 0.48, *p* < 1 × 10^−16^). The most prominent change in connectivity for non-hub to hub status was seen for *Ntrk2* (0.197 vs. 0.821; Low vs. High line). *Nrtk2* encodes TrkB, a receptor for Bdnf.

GO annotation of the SH DV genes revealed a significant enrichment in genes associated with cell-to-cell signaling (FDR < 2 × 10^−4^; **Supplementary Table S6**) and neuron projection (FDR < 7 × 10^−3^). The signaling genes (*N* = 33) in addition to the common DV genes noted above included C*hat, Chrna7, Grik2, Grin2b Htr1b, Htr2a, Oprd1, Sv2c*, and 10 protocadherins. The SH DV genes were enriched (*p* < 2 × 10^−21^) in a single network module, magenta; 187 or the 231 members of the magenta module were significantly DV between the High and Low selected lines. The magenta module was enriched in genes associated with cell-to-cell signaling (*p* < 3 × 10^−11^) and neuron part (*p* < 4 × 10^−5^; **Supplementary Table S6**). The magenta module signaling genes overlapped with those noted above, e.g., *Chrna7* and *Grin2b*, and included 12 protocadherins. Focusing on the DV genes within the magenta module, average intramodular connectivity increased from 0.34 to 0.73 (Low vs. High; *p* < 1 × 10^−63^). Genes showing large changes (non-hub to hub status; Low vs. High) included *Pde4d, Adra1a, Pcdhga8&6*, and *Ncam2*.

GO annotation of the PL DV genes revealed a significant enrichment in genes associated with signal transduction (FDR < 1 × 10^−6^), cell to cell signaling (FDR < 1 × 10^−4^) and neuron part (FDR < 8 × 10^−9^) (**Supplementary Table S6**). The signaling genes overlap with those noted above but also include *Nos1* and *Gm3*. The PL DV genes were enriched (*p* < 3 × 10^−10^) in a single network module, brown. GO annotations for the brown module included neuron part (FDR < 5 × 10^−11^), PDZ domain signaling (FDR < 1 × 10^−2^) and cell to cell signaling (FDR < 7 × 10^−11^). The signaling genes (*N* = 49) largely overlap the signaling genes in the blue and magenta modules noted above and include a large number (*N* = 18) of protocadherins, also seen prominently in the SH results. Focusing on the brown module DV genes, average intramodular connectivity differed between the High vs. Low lines (0.72 vs. 0.40; *p* < 5 × 10^−20^). Genes showing large differences in hub status (High > > Low) included *Syt10, Dgkh, Grin2a*, and *Adra1a*.

### Differential Wiring (DW) Across Regions

There were 1,392, 1,445, and 879 genes showing significant (adjusted *p-*value < 0.05) DW between the High and Low selected lines in the CeA, SH, and PL, respectively (**Supplementary Table S7**). The overlap in DW is illustrated in **Figure 3D**. Seventy-two genes were common to all three regions and this grouping was significantly (FDR < 5 × 10^−3^) enriched in genes associated with the post-synaptic component. Genes with this GO annotation included *Chrna7, Als2, Pppir9a, Strn, Kcna4, Kif1a*, and *Slc1a2* (**Supplementary Table S8**). The overlap in genes between the PL and SH (*N* = 123) showed a significant (FDR < 2 × 10^−2^) enrichment in genes with the synaptic membrane annotation; these genes included *Arrb1, Itgb1, Cpd, Akap5, Rim1, Shank3, Ptprz1, Gm3, Ank2, Gm1*, and *Cntmap2.* There was no annotation enrichment in the overlapping genes between the PL and CeA. The overlapping genes between the CeA and the SH (*N* = 309) showed a significant (FDR < 2 × 10^−3^) enrichment in genes associated with the neuronal component (**Supplementary Table S8**); genes in this category (*N* = 60) included *Calm1, Gad2, Nlg1, Oprd1, Pten, Rab10*, and *Sv2a.*

GO annotation of the CeA DW genes revealed a significant enrichment (FDR < 4 × 10^−7^) in genes (*N* = 118) associated with the synaptic component (**Supplementary Table S8**); genes in this category included *Cnr1, Dlg1, Gabra4, Gabrb3, Gabrg3, Gphn, Gria2, Grid2, Grik3, Grin2b, Grm5, Slc1a2&3, Stx1b,2&3*, and *Syap1*. The CeA DW genes were enriched in three modules: blue, magenta, and turquoise (**Supplementary Table S8**). The blue CeA module is described above. The magenta module did not have a significant enrichment in any GO category. The turquoise module was enriched in the categories macromolecule metabolic process (FDR < 1 × 10^−8^), ubiquitin–protein transferase activity (FDR < 2 × 10^−4^) and membrane-bound organelle (FDR < 1 × 10^−9^). It also should be noted that for six modules, the number of DW genes was significantly less than expected; these modules were significantly conserved in response to selection. For the DW genes in the blue, magenta, and turquoise modules, intramodular connectivity (Low vs. High; 0.26 vs. 0.55) was significantly different (*p* < 1 × 10^−95^). Large changes (>0.5 in relative connectivity) were noted for the genes *Cab39, Nrtk2, Ankrd10*, and *Mov10;* all of these genes increased relative connectivity from the Low to the High line. Additional details for *Nrtk2* are noted above.

GO annotation of the SH DW genes revealed a significant enrichment (FDR < 4 × 10^−12^) in genes (*N* = 137) associated with the synaptic component (**Supplementary Table S8**); genes in this category and not noted previously included *Adam10, Arrb1, Epha4, Grm4&7, Homer1, P2ry1, Snap25&29, Synpo*, and *Synpr.* The SH DW genes were enriched in five network modules, most prominently in the green module (*p* < 4 × 10^−9^). The green module was significantly enriched in genes associated with the synaptic component (*p* < 2 × 10^−8^), nervous system development (*p* < 1 × 10^−4^) and enzyme binding (*p* < 2 × 10^−2^). For nine modules, the number of DW genes was significantly less than expected. For the green module DW genes, intramodular connectivity on average showed no change between the Low and High lines (0.61 vs. 0.62, respectively).

GO annotation of the PL DW genes revealed a significant enrichment in genes (*N* = 75) associated with the synaptic component (FDR < 2 × 10^−8^) and in genes (*N* = 282) associated with development (FDR < 2 × 10^−4^; **Supplementary Table S8**). Genes in the synapse category and not noted previously included *Cadm1&2, Dmd, Kcna2&4, Phactr1, Snph, Sntb1, and Tln1.* The PL DW genes were significantly enriched in two network modules, brown and green. The brown module is described above. The green module was enriched in genes associated with regulation of cellular localization (FDR < 2 × 10^−2^) and in genes associated with the neuronal component (FDR < 6 × 10^−4^). Different from the CeA and SH, only one PL module (yellow) showed significant conservation (corrected *p* < 0.05). For the brown module DW genes, relative intramodular connectivity increased in the High vs. Low line (0.59 vs. 0.24; *p* < 6 × 10^−56^). Genes moving from non-hub status (Low line) to hub status (High line) included *Sox6, Egr3, Soga3, Pcdhgb5, Pcdhga8, Senp5*, and *Prkg1*. For the green module DW genes, relative intramodular connectivity increased in the High vs. Low line (0.52 vs. 0.21; *p* < 2 × 10^−37^). Genes moving from non-hub status (Low line) to hub status (High line) included *Ncoa4, Edem3, Xpr1*, and *Necab1*.

## Discussion

We recognize that there are many strategies available for analyzing complex datasets, such as those presented here, and each will emphasize somewhat different aspects of the data. The approach taken here is one that we have used previously (Colville et al., 2017; Iancu et al., 2018). The key metrics; DE, DV, and DW, are computationally straightforward and can be easily replicated. The WGCNA has greatly matured since its introduction (Zhang and Horvath, 2005) and has been used in more than 300 publications. In the current study we have focused our investigations on those genes that contribute to at least 80% of network connectivity. This thresholding reduced the number of genes considered for further analyses from ∼15,000 to ∼6,500 in each of the three brain regions. The genes culled are “leaf” nodes with low connectivity. While selection will have significant effects on some of these culled genes, none will be hub nodes. We also note that the sample sizes used in the current study were sufficient to produce networks of high quality (Langfelder and Horvath, 2008). The selection of the High and Low ethanol preference lines from HS-CC founders has been described elsewhere (Colville et al., 2017). The HS-CC was derived from eight mouse strains, including three wild-derived strains; the genetic diversity captured is ∼90% of that available in *M.* *musculus* (Roberts et al., 2007). The preference lines were bred using a short-term selective breeding protocol (Belknap et al., 1997; Metten et al., 2014) that minimizes the stochastic fixation of alleles unrelated to the phenotype of interest, here 2-bottle choice ethanol preference. From the perspective of ethanol preference and consumption, the HS-CC are of interest in that ∼25% of the animals show a preference for ethanol; this differs from a <5% preference found in our HS/NPT mice (unpublished observation) that were derived from eight laboratory mouse strains (Hitzemann et al., 2014).

Contet (2012) surveyed the existing literature and noted that multiple functional categories were associated with a “predisposition” to excessive ethanol consumption and in most cases each of the categories have been supported by multiple publications (see Table 2 in Contet, 2012). Some regional specificity for each of the functional categories was also noted; however, the regional differences in gene expression were generally larger than those associated with selection for preference or binge drinking (Kimpel et al., 2007; Mulligan et al., 2011). Subsequent studies have confirmed and extended the “region” effect (e.g., Melendez et al., 2012; Osterndorff-Kahanek et al., 2015; Smith et al., 2016; Mulligan et al., 2017). The data in **Supplementary Table S1** again confirm marked differences in regional gene expression. Fifty or more genes in each of the three regions show a 10-fold higher expression when compared with at least one other region. In no region was selection associated with a change in expression of >2-fold and in most cases, selection was associated with small changes in expression (<30%) among the genes included in the DE analyses (see above). The number of significantly DE genes, common to all three regions was small (*N* = 5) and the genes appear to have no common function(s). Only in the CeA, did the analyses reveal that the DE genes were associated with significant GO annotations (neuron part, structural constituent of myelin sheath and axon ensheathment). Among the genes in the neuron part category were several that have been implicated in excessive ethanol consumption, including *Adora1, Crhr1, Gal*, and *Syt2* (Belfer et al., 2006; Enoch et al., 2013; Barbier et al., 2015; Clark et al., 2017). However, in the CeA as well as the SH and PL, the DE genes had on average a low intramodular connectivity, i.e., these genes were “leaf” nodes. This observation is consistent with the observation that the degree of DE was generally quite small and to detect such small changes requires that the variance for these genes must be relatively low. Connectivity requires sufficient variance to accurately detect gene–gene correlations (see below). Overall, we conclude that DE is not a key selection feature for preference lines derived from genetically diverse HS-CC founders and when viewed in a network context. A similar conclusion was reached on a smaller SH sample (Colville et al., 2017).

The relationship(s) between network connectivity and gene variability are not entirely clear. However, if the variance is “biological” and not technical or simply stochastic, it follows that variance and connectivity will increase in tandem; for the moderate sample sizes of most gene expression studies, gene–gene correlations and hence connectivity will be more easily detected. Colville et al. (2017) observed that selection for the High and Low preference lines was associated with a cluster of DV genes that were highly enriched in a single network module (greenyellow). The module was highly enriched in genes associated with receptor signaling (e.g., *Chrna7*, *Grin2a*, *Htr2a*, and *Oprd1*) but also included a large number of genes associated with cell adhesion. Cadherins and protocadherins were particularly enriched in the greenyellow module. Expanding the SH sample size from Colville et al. (2017) by ∼50% did not perceptually change the results. In the SH, the DV genes were highly enriched in a single module (magenta) that was similar to the greenyellow module (again remembering that module color has no meaning and is randomly assigned). The magenta and greenyellow modules are of a similar size (231 vs. 227 genes, respectively); 98 genes overlap between the modules (**Supplementary Table S9**). The modules share 37 hub nodes; including *Oprd1, Dlg2, Gabrb2, Pcdhgb2, Pcdhga6, and Pcdhga7*, i.e., a measure of core connectivity is unchanged. The differences between the modules are largely found in the less connected nodes.

The CeA and PL DV genes also were enriched in single network modules, blue and brown, respectively. Annotation of these modules was similar to that for the SH magenta module, e.g., a significant enrichment in genes associated with cell to cell signaling. The SH magenta, the CeA blue and the PL brown modules were significantly different in size (231, 773, and 593 genes, respectively). However, 183 (79%) the genes in the SH module are also found in the CeA and PL modules. This grouping of module core genes is found in **Supplementary Table S9**. This core grouping contains several receptors including *Adra1a, Chrna7, Grin2b, Htr2a, Oprd1*, and *Sstr4;* this core group also contains 17 protocadherins including 14 of the 22 known γ protocadherins. Thirty significant DV genes were identified as common to all three regions (see **Figure 2**); 25 genes of this group are found in the core module grouping. Within the core module grouping, we identified the 18 genes that were hub nodes across all three regions; we next aligned these genes with the 25 common DV genes found in the core module. Our rationale for this step was to identify high priority hub nodes, that could be targeted in future studies. Six genes were identified: *Dlg2, Gatad2b, Pcdhac2, Tnks, Usp29*, and *Usp9x.* *Dlg2* encodes for post-synaptic density protein 93 (PSD-93), *Gatad2b* encodes for transcriptional repressor p66-beta, *Pcdhac2* encodes for protocadherin αc2, *Tnks* encodes for Tankyrase-1, *Usp29* encodes for ubiquitin specific protease 29 and *Usp9x* encodes for ubiquitin specific protease 9, X-linked. That two ubiquitin-related genes are in this group cannot be unexpected, given the long standing observations that ubiquitination is associated with chronic ethanol exposure in both animals and humans (see Sokolov et al., 2003; Liu et al., 2006; Contet, 2012; Melendez et al., 2012; Widagdo et al., 2017). Our data link ubiquitination to risk for excessive consumption. The precise mechanisms are unknown but we note here that ubiquitination has a key role in glutamate receptor trafficking (Widagdo et al., 2017). The functions of Tankyrase-1 (Tank-1) in the brain have not been investigated. However, Tank-1 is a member of a large family of poly (ADP-ribose) polymerases (PARPs). PARP-1 is thought to have key role(s) in the neuroinflammatory cascade associated with binge ethanol consumption (Tajuddin et al., 2018). To our knowledge, Pcdhαc2 has no function remarkably different from the other members of the αPcdh family; however relatively little is known about functions of the individual gene products. What the data presented previously (Colville et al., 2017) and again confirmed here clearly illustrates that selection for ethanol preference engages a large number of the clustered protocadherins. Again with a focus on glutamate neurotransmission, Suo et al. (2012) have shown that both the α and γ protocadherin clusters are involved in the inhibition of Pyk2 (protein tyrosine kinase 2), which results in the disinhibition of Rac1 (Ras-related C3 botulinum toxin substrate 1) that in turn can facilitate the proper assembly of dendritic spines (see Figure 8 in Suo et al., 2012). Mutations and deletions in *Gatad2b* have been associated with intellectual disabilities (e.g., Tim-Aroon et al., 2017). Perhaps more pertinent for the current study, the ENIGMA consortium has found that SNPs associated with both *Gatad2b* and *Dlg2* are associated with differences in putamen size (Chen et al., 2017). The coexpression and physical interaction partners for *Dlg2* are shown in **Figure 1**. Key partners include a number of genes encoding glutamate receptor subunits (e.g., *Grin2b* and *Grid1*) and genes encoding glutamate associated membrane proteins (e.g., *Dlg1, Dlg4*, and *Dlgap1*). Bell et al. (2016) have reviewed the literature associated with ethanol risk, ethanol effects and glutamate reward circuitry; importantly, these authors noted when comparing the P and NP rats, there were a number of differences in glutamate signaling genes that predate ethanol exposure. Clinical studies have shown that in family history positive (FHP) individuals there is an altered response to both alcohol and the NMDA antagonist ketamine, suggesting a genetic link between alcoholism and NMDA receptor function (Petrakis et al., 2004; Joslyn et al., 2010).

Differential wiring which is necessarily related to DV, provided a measure of how selection affects the interaction (connectivity) of each gene with the entire coexpression network. Similar to our previous results (Colville et al., 2017; Iancu et al., 2018), we observed that selection has marked effects on DW and this was true across all regions, with the effects somewhat more prominent in the CeA and SH than the PL. The large DW effect associated with selection is largely silent in most analyses of coexpression data, even though the data illustrate here that the rewiring of the coexpression system is perhaps the most profound change in the transcriptome. There were 71 common DW genes across the three brain regions and this core group was significantly enriched in genes associated with the post-synaptic membrane. The genes in this category included *Chrna7, Als2, Ppp1r9a, Strn, Kcna4, Kif1a, and Slc1a2.* *Slc1a2*, which encodes for the excitatory amino acid transporter 2 (EAAT2) and is the principal transporter within the brain for glutamate, is of interest given the focus on excitatory neurotransmission above and evidence that inhibition of EAAT2 reduces ethanol consumption (Sari et al., 2016). Other members of this group appear to have some role(s) in regulating glutamatergic receptor function. For example, the deletion of *Chrna7* leads to the loss of NMDA receptors (Lin et al., 2014). Interestingly, the deletion of *Chrna7* is also associated with increased sensitivity to several ethanol-induced behaviors (Bowers et al., 2005). *Als2* encodes for alsin which has been shown to protect neurons from glutamate-associated neurotoxicity (Lai et al., 2006; Kwak and Weiss, 2006; Cai et al., 2008). *Strn* which encodes for striatin, is highly enriched in dendritic spines; this localization is reduced by NMDA receptor stimulation which appears to have a key role in synaptic plasticity (see Chen et al., 2012 and references therein). *Kcna4* which encodes potassium voltage-gated channel subfamily A member four, is recruited to the synapse by PSD95, where it is phosphorylated (Wong and Schlichter, 2004). *Kif1a* encodes a kinsin family three member which is also known as axonal transporter of synaptic vesicles. Mutations in the *Drosophila* homolog unc-104, have revealed the importance of the protein product in glutamate spontaneous release and in post-synaptic density organization (Zhang et al., 2017).

In each of the three brain regions, the DW genes unique to that region were highly enriched in synapse-associated genes. This effect was particularly dramatic in the CeA where a large number of both GABA and glutamate receptor subunits were affected. DW genes were distributed across several network modules, making the distribution of the DW genes more diffuse than that for the DV genes. It was also observed in both the CeA and SH that a number of network modules were largely preserved from the effects of selection on wiring. Many of these preserved modules had annotations associated with ATP metabolic processes, DNA replication, rRNA cellular respiration and so on. One purpose of using a short-term selective breeding protocol is to minimize genetic drift and focus the analysis on only those alleles associated with the phenotype of interest, here ethanol preference. Clearly, the DW data illustrates that even three rounds of selection had marked and extensive effects on the brain transcriptome.

Our discussion has largely focused on those changes in gene expression that are similar across the three brain regions. Our argument for taking this perspective is that these changes are the “broad” targets for manipulation. Included in these broad targets are core genes, including hub nodes, associated with glutamate receptor signaling and synaptic plasticity. We have also confirmed (see Colville et al., 2017) that selection for ethanol preference in HS-CC mice involves a large cohort of clustered protocadherins. This differs from selection for binge ethanol consumption where we have observed that selection for “drinking in the dark” involves numerous extra-cellular matrix genes such as collagens and matrix metalloproteases (Iancu et al., 2018). “Narrow” sense targets for manipulations will include those selection based changes that are regionally unique. For example, we observed that in the CeA, the expression of *Nrtk2* which encodes TrkB, a receptor for Bdnf, moves from non-hub status in the Low selected line to hub status in the High line. Numerous studies have linked the regulation of ethanol consumption to the regulation of Bdnf function; Darcq et al. (2016) have found in the rat dorsolateral striatum the Bdnf-TrkB system is essential to maintaining moderate ethanol intake. Our data suggest that manipulating this system in the CeA will likely have marked effects on ethanol preference.

## Author Contributions

AC performed all of the dissections and RNA extractions as well as the post-sequencing data analysis and preparation of the manuscript. OI conducted data analysis and manuscript and figure preparation. DL performed the behavioral selection to derive the mice used and helped prepare the manuscript. PD contributed to data analysis. SM contributed to experimental design, data analysis, and manuscript preparation. RS performed the sequencing and contributed to experimental design. CZ contributed to data analysis. RH was the PI for this project and oversaw all components, also contributing to data analysis and manuscript preparation.

## Conflict of Interest Statement

The authors declare that the research was conducted in the absence of any commercial or financial relationships that could be construed as a potential conflict of interest. The reviewer RB and handling Editor declared their shared affiliation.

## Footnotes

1 http://www.ncbi.nlm.nih.gov/geo/query/acc.cgi?acc=GSE65950

## Supplementary Material

TABLE S1 Gene expression (RNA-Seq) in the CeA, SH, and PL expressed as counts (reads) per million (CPM) – average data (*N* = 67, 71, and 54, respectively).

TABLE S2 Annotation of how the network modules in each of the brain regions were significantly enriched in genes associated with neurons, astrocytes, and oligodendrocytes.

TABLE S3 Differential expression (DE) between the High and Low selected lines in the CeA, SH, and PL (FDR < 0.05).

TABLE S4 Gene Ontology (GO) annotation for DE genes.

TABLE S5 List of genes found to be differentially variable (DV) in the CeA, SH, and PL (FDR < 0.05).

TABLE S6 GO annotation of overlapping DV genes between brain regions.

TABLE S7 List of differentially wired (DW) genes in the CeA, SH, and PL (FDR < 0.05).

TABLE S8 GO annotation of overlapping DW genes between brain regions.

TABLE S9 Comparison of data taken from this study with that of previous work from our laboratory.
